# Digital evaluation of nasal changes induced by rapid maxillary expansion with different anchorage and appliance design

**DOI:** 10.1186/s12903-017-0404-3

**Published:** 2017-07-14

**Authors:** Rosamaria Fastuca, Paola Lorusso, Manuel O Lagravère, Ambra Michelotti, Marco Portelli, Piero Antonio Zecca, Vincenzo D’ Antò, Angela Militi, Riccardo Nucera, Alberto Caprioglio

**Affiliations:** 1 Department of Surgical and Morphological Sciences, University of Messina, Via Consolare Valeria 1, Messina, Italy; 2 Private Practice in Orthodontics, Bari, Italy; 3 Research Fellow, University of insubria, Via G. Piatti, 10 Varese, Italy; 4 Department of Dentistry, University of Alberta, 11400 University Avenue, Edmonton, AB Canada; 5 Section of Orthodontics, Department of Neuroscience, Reproductive Sciences and Oral Sciences, University of Naples Federico II, Via Pansini, 5, Naples, Italy; 6 Department of Biomedical Sciences, Dentistry and Morphological and Functional Imaging, University of Messina, Via Consolare Valeria 1, Messina, Italy; 7 Department of Surgical and Morphological Sciences, School of Medicine, University of Insubria, Via G. Piatti, 10 Varese, Italy; 8 School of Orthodontics, University of Naples, Naples, Italy; 9 Department of Biomedical Sciences, Dentistry and Morphological and Functional Imaging, University of Messina, Via Consolare Valeria 1, Messina, Italy; 10 Department of Biomedical Sciences, Dentistry and Morphological and Functional Imaging, University of Messina, Via Consolare Valeria 1, Messina, Italy; 11 Department of Surgical and Morphological Sciences, University of Insubria, Via G. Piatti, 10 Varese, Italy; 12 C/O Dental School, Via G. Piatti, 10, 21100 Velate, VA Italy

**Keywords:** Rapid maxillary expansion, Nasal cavity, Low dose computed tomography, Cone beam computed tomography

## Abstract

**Background:**

Scientific evidence showed that rapid maxillary expansion (RME) affects naso-maxillary complex, increasing nasal width and volume. This study aimed to evaluate nasal changes induced by rapid maxillary expansion with different anchorage and appliance design by using low dose and cone beam computed tomography.

**Methods:**

A total of 44 patients (20 males, mean age 8y 8 m ± 1y 2 m; 24 females mean age 8y 2 m ± 1y 4 m) were included in the investigation and divided into three groups according to the appliance: Hyrax-type expander anchored to permanent teeth, modified Hyrax-type expander anchored to deciduous teeth, modified Haas-type expander anchored to deciduous teeth. Maxillary expansion was performed until overcorrection and the expander was passively kept in situ for 7 months at least. All patients had three-dimensional imaging before expansion (T0) and after the retention period (T1). Nasal floor width, nasal wall width, maxillary inter-molar width were measured by means of Mimics software. The paired sample t-test was employed to assess the significance of the differences between the time points; the analysis of variance test (ANOVA) was used to compare differences between groups.

**Results:**

The statistical analysis revealed significant differences between T0 and T1 for each recorded measurement in each group; no significant differences were found by comparing groups.

**Conclusions:**

Rapid maxillary expansion produces a significant skeletal transverse expansion of nasal region in growing patients. No significant differences in nasal effects are expected when the appliance is anchored onto deciduous teeth, with or without the palatal acrylic coverage.

## Background

Rapid maxillary expansion (RME) represents a routine orthodontic procedure aimed to increase maxillary transverse dimensions in growing patients. The effects of RME on craniofacial structures have been extensively studied in the literature [1, 2] and are referred not only to anatomical structures close to the maxillary bone, but also to cranial base and temporomandibular joint [3]. Several authors suggested that RME affected the whole naso-maxillary complex, increasing nasal width and volume, also reducing the resistance of nasal airflow [4–6]. However, scientific evidence does not seem to recommend RME for the sole purpose of restoring the respiratory function [1]. RME is achievable by means of various appliances and treatment protocols, including the most recently developed bone-anchored expansion [7, 8]. The most common RME procedure is performed with tooth-borne or tooth-tissue-borne palatal expanders [9, 10]. Usually, the appliance is anchored to the upper permanent first molars, which may exhibit undesirable side effects including root resorption [11], buccal tipping, gingival recession and bone loss [12]. In order to avoid the abovementioned effects on the permanent supporting teeth, some authors proposed a modified expander anchored to deciduous teeth [13, 14]. Although deciduous teeth anchorage in RME therapy is becoming more popular among clinicians, there is a lack of investigations concerning the maxillary effects of these appliances [12–17] and a little recent scientific evidence of their impact on the nasal cavities [18–20]. In the last decades considerable advances in three-dimensional imaging techniques and related software were achieved, contributing to extend the possibilities in orthodontic diagnosis, treatment and follow-up [21–24]. Low dose computed tomography (CT) is a well-accepted tool for the morphologic evaluation of the craniofacial complex [21] and it was proposed to examine nasal cavities [25, 26]. Recent evidence also reported the use of cone beam computed tomography (CBCT) for analysing upper airway [18, 27]. Low overall radiation dose, low cost, accessibility to dentist, accuracy and reliability are some of the advantages of CBCT claimed by investigators [5, 28, 29]. The purpose of this retrospective study was to assess nasal changes induced by RME with different anchorage and appliance design by using low dose CT and CBCT.

## Methods

The initial sample of the present retrospective study consisted of 163 patients treated with RME, selected from the Departments of Orthodontics of University of Insubria (Varese, Italy) and University Federico II (Naples, Italy). Signed informed consent for releasing diagnostic records for scientific purposes was available from parents of patients. Protocol was reviewed and approved by the Ethical Committee (Approval n^o^. 826) and procedures followed adhered to the World Medical Organization Declaration of Helsinki. Sample size was calculated on the measurements of two patients per group selecting as main outcome the maxillary inter-molar width changes before and after treatment. A sample size of at least 10 subjects per group was necessary to detect a power of 0.8. Among all patients only who satisfied inclusion and exclusion criteria were selected for the final group. The inclusion criteria were as follows: good general health, early mixed dentition, stage 1 or 2 of cervical vertebral maturation (CVM), transverse maxillary deficiency with unilateral or bilateral cross-bite treated by using RME, availability of complete initial and final records including CT scans, photographs, dental casts, medical history forms.

Exclusion criteria comprised systemic diseases and craniofacial syndromes [30–32], severe facial asymmetry [33, 34], dental anomalies, naso-pharyngeal diseases, history of nasal or tonsil surgery, age above 15 years, stage 3 or more of CVM, history of other orthodontic treatment prior to RME. From the initial sample of 163 patients, 44 patients (20 males, mean age 8y 8 m ± 1y 2 m; 24 females mean age 8y 2 m ± 1y 4 m), treated between January 2013 and September 2015, were included in the study. The selected patients were divided into three groups according to the type of appliance used to perform RME:

- Hyrax-type expander anchored to permanent teeth (HX-6) – 15 patients
- Modified Hyrax-type expander anchored to deciduous teeth (HX-E) – 14 patients
- Modified Haas-type expander anchored to deciduous teeth (HS-E) – 15 patients

**Fig. 1:**
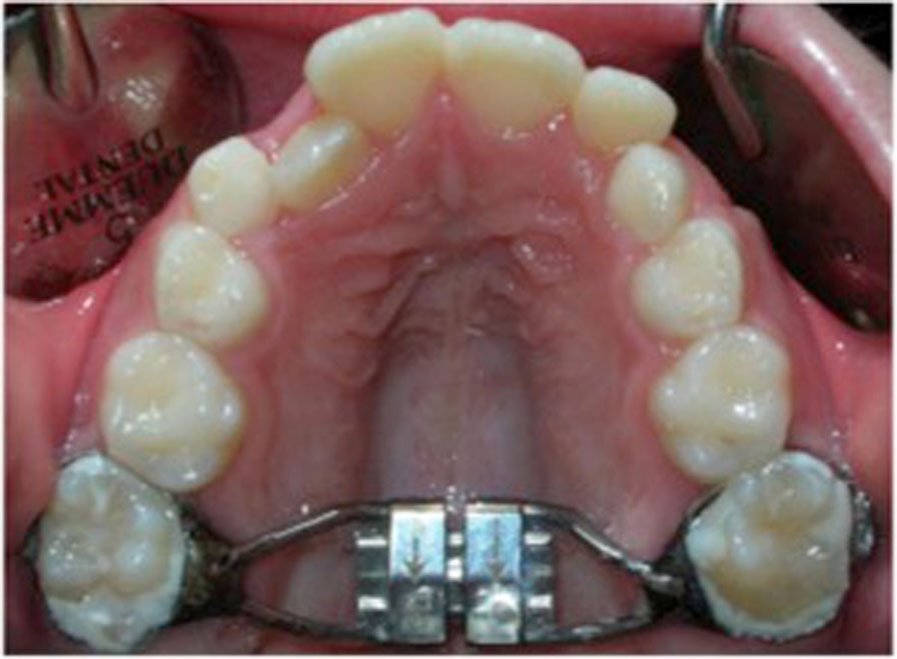
Hyrax-type expander anchored to permanent teeth

The subjects of HX-6 group were treated with a traditional Hyrax-type expander (Leone Orthodontics Products, Sesto Fiorentino, Firenze, Italy) banded to the upper permanent first molars (Fig. 1).

In HX-6 group the screw of the expander was initially turned eight times; afterwards the parents of the patients were instructed to turn the screw three times a day [35].

**Fig. 2:**
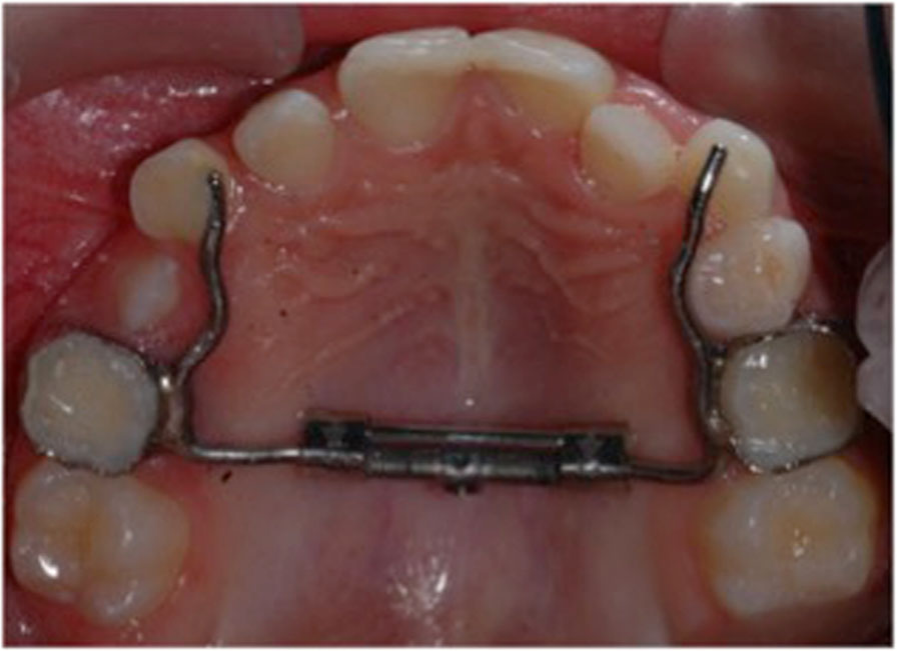
Modified Hyrax-type expander anchored to deciduous teeth

In HX-E group RME was accomplished by using a Hyrax-type expander (Leone Orthodontics Products, Sesto Fiorentino, Firenze, Italy) modified to be anchored to the upper deciduous second molars with cemented bands and provided with extensions of the metal framework up to the deciduous canines (Fig. 2).

**Fig. 3:**
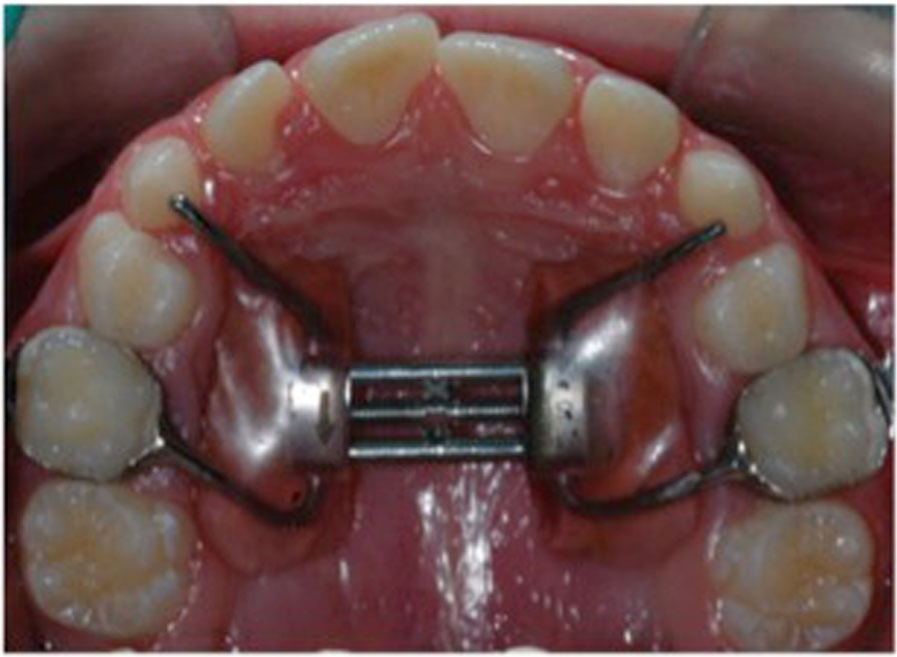
Modified Haas-type expander anchored to deciduous teeth

The patients of HS-E group were treated by means of a Haas-type expander (A167–1439, Forestadent, Pforzheim, Germany) modified with bands cemented to the upper deciduous second molars and with anterior arms bonded to the upper deciduous canines (Fig. 3).

In both groups, HX-E and HS-E, the screw was initially activated twice by the clinician; after that, it was turned once or twice per day by the parents of the patients. Each activation was equal to 0.20 mm for the devices of HX-6 and HX-E groups, to 0.225 mm for the expanders of HS-E group. Maxillary expansion was performed until the dental overcorrection (the lingual cusps of the upper permanent first molars occlude onto the buccal cusps of the lower ones) was achieved. When dental overcorrection was clinically observed, the screw was stabilized and the expander was passively kept in situ as a retainer to allow the bone formation in the expanded midpalatal suture. The retention period lasted at least 7 months.

In order to evaluate nasal and maxillary widths the three-dimensional imaging records of patients were used. All patients selected for the study had low dose CT or CBCT imaging before expansion (T0) and when the expander was removed (T1). CT examinations were performed by means of the same CT scanner (MX 8000 IDT6, Philips Medical Systems, Best, The Netherlands) using a low dose protocol (KV 80, mAs 28, Pitch 1, CDTIVol 2.5 mgy), with the patient in supine position. CBCT images were taken by means the same CBCT scanner (i-CAT, Imaging Sc. Int., Hatfield, PA, U.S.A.) using unchanged setting parameters (120 KV, 3.8 mA, 30 s) with the patient in seated position and the head in the natural head position [36]. The DICOM files were processed in Mimics software (version 10.11, Materialise Medical Co, Leuven, Belgium). A set of landmarks was identified in order to obtain the planes for a reproducible position of the head and to compare the images between the pre and post-treatment examination. The head orientation allowed for the comparisons of different patients without risking of measurements deformations. The landmarks used included: anterior and posterior nasal spine (ANS and PNS), right and left foramen spinosum (RFS and LFS). The derived planes were: plane passing through ANS and PNS, plane passing through the bilateral FS. The definition of the landmarks is reported in Table 1.

**Table 1:** Skeletal landmarks for image reslicing

Landmarks	Definitions
ANS	The most anterior point of the anterior nasal spine
PNS	The most posterior point of the posterior nasal spine
RFS	The geometric center of the smallest circumference with defined borders view axially on the right foramen spinosum
LFS	The geometric center of the smallest circumference with defined borders view axially on the left foramen spinosum

*ANS* anterior nasal spine, *PNS* posterior nasal spine, *RFS* right foramen spinosum, *LFS* left foramen spinosum

**Fig. 4:**
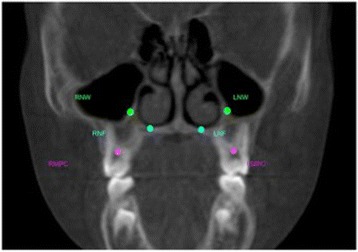
Dental and skeletal landmarks on a CBCT coronal scan. RMPC, right molar pulp chamber; LMPC, left molar pulp chamber; RNF, right nasal floor; LNF, left nasal floor; RNW, right nasal wall; LNW, left nasal wall

After reorienting images, a second set of reproducible dental and skeletal landmarks was identified. All the landmarks were located in the same coronal scan passing through a stable dental point corresponding to the first upper right molar furcation (RMF). All the landmarks are reported in Table 2 and Fig. 4.

**Table 2:** Set of reproducible dental and skeletal landmarks located on the scans

Dental landmarks	Definitions
RMPC	Center of the pulp chamber of the first upper permanent molar of the right side
LMPC	Center of the pulp chamber of the first upper permanent molar of the left side
Skeletal landmarks	Definitions
RNF	Junction of palatal cortical alveolar bone and cortical bone surrounding nasal cavity of the right side located in the coronal scan passing through RMF point
LNF	Junction of palatal cortical alveolar bone and cortical bone surrounding nasal cavity of the left side located in the coronal scan passing through RMF point
RNW	Most external point of the cortex bone separating the maxillary sinus and the nasal cavity of the right side located in the coronal scan passing through RMF point
LNW	Most external point of the cortex bone separating the maxillary sinus and the nasal cavity of the left side located in the coronal scan passing through RMF point

*RMF* right molar furcation, *RMPC* right molar pulp chamber, *LMPC* left molar pulp chamber, *RNF* right nasal floor, *LNF* left nasal floor, *RNW* right nasal wall, *LNW* left nasal wall

**Fig. 5:**
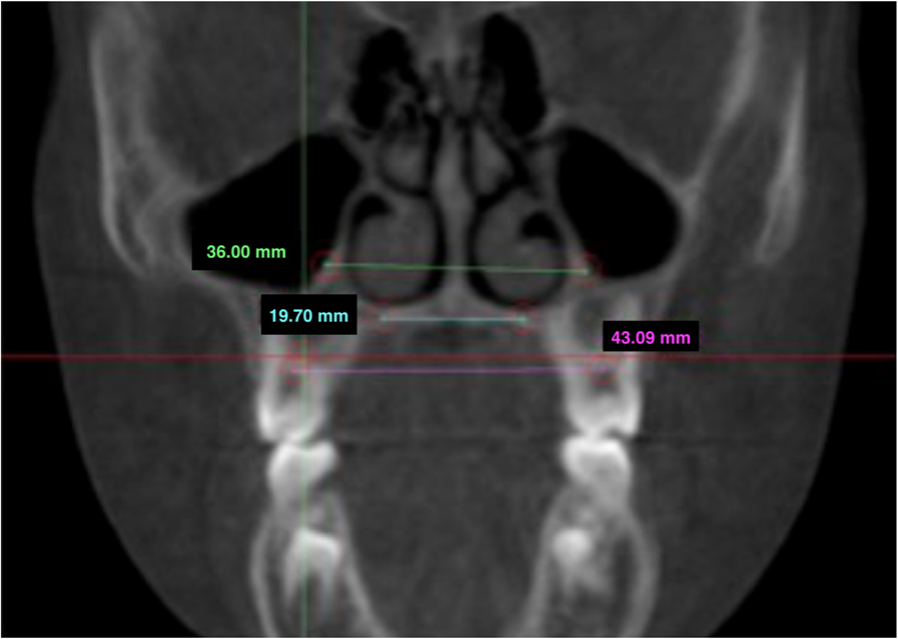
Linear transverse measurements performed by using Mimics software

Using the above mentioned landmarks, a set of linear transverse measurements was performed as shown in Table 3 and Fig. 5.

**Table 3:** Variables tested in HX-6, HX-E and HS-E group

Variables (mm)	Definitions
Maxillary inter-molar width	RMCP to LMCP
Nasal floor width	RNF to LNF
Nasal wall width	RNW to LNW

*RMCP* right molar pulp chamber, *LMPC* left molar pulp chamber, *RNF* right nasal floor, *LNF* left nasal floor, *RNW* right nasal wall, *LNW* left nasal wall

The landmark location and the transverse measurements were manually performed by two investigators. In order to minimize the method error, 9 patients (3 for each group) were randomly selected and their images were resliced and measured again 1 month later. No significant differences between the two series of records were found by using paired sample t-tests. Error between the two different x-rays techniques was calculated a priori by comparing the measurements on the x-rays to the measurements on plaster models of the same patient. The measurement was performed on the mesio-distal width of the first upper right permanent molar of five randomly selected patients for CT scan and ten for the CBCT scan. The correlation between the two measurements was evaluated with Pearson correlation coefficient and it resulted of 0.91 for CT scan and 0.90 for CBCT scan. The difference between the two x-ray technique was then considered not significant.

Means and standard deviations (SDs) were calculated for the measurements understudy (maxillary inter-molar width, nasal floor width, nasal wall width) at T0 and T1 in each group. Statistical analysis was performed by means of a software (MedCalc Software - Version 11.5.1.0, Mariakerke, Belgium). Parametrical methods were used after having tested the existence of the assumptions through the Shapiro-Wilk test for the normality of the distributions and through Levene test for the equality of variances. The paired sample t-test was employed to assess the significance of the differences of each measurement between the time points within each group. A *p*-value less than 0.05 was used in the rejection of the null hypothesis, i.e. no significant difference exists in the measurements understudy between T0 and T1. The analysis of variance test (ANOVA) was used to compare the differences T1-T0 of each measurement between groups. Bonferroni correction was applied for statistically significant differences after post–hoc analysis. The second null hypothesis of this investigation stated that there was no difference among the three different expanders understudy in the effects on the nasal cavities.

## Results

Comparison of the starting forms showed no significant differences in the examined variables, then indicating that the groups were comparable. The T-test analysis revealed statistically significant differences between T0 and T1 for each recorded measurements in each group (Table 4), showing that all types of expander produced a significant increase of the dental and nasal skeletal transverse measurements understudy.

**Table 4:** Paired samples t-test for comparisons between the time points T0 and T1 for each variable in each group

Variables	Groups	T0	T1	
		Mean ± SD	Mean ± SD	p^a^
Maxillary inter-molar width (mm)	HX-6	37.56 ± 1.37	42.90 ± 2.85	0.00014*
	HX-E	41.98 ± 3.33	45.69 ± 3.14	0.00040*
	HS-E	39.42 ± 3.55	43.76 ± 3.22	0.00095*
Nasal floor width (mm)	HX-6	18.79 ± 2.36	21.84 ± 3.12	0.00004*
	HX-E	19.07 ± 2.71	22.17 ± 3.02	0.00138*
	HS-E	17.13 ± 4.18	20.03 ± 3.97	0.00478*
Nasal wall width (mm)	HX-6	26.01 ± 1.79	28.37 ± 3.04	0.00019*
	HX-E	29.17 ± 1.90	31.62 ± 2.73	0.00017*
	HS-E	27.80 ± 3.01	30.47 ± 2.12	0.00622*

*HX-6* Hyrax-type expander anchored to permanent teeth, *HX-E* Hyrax-type expander anchored to deciduous teeth, *HS-E* Haas-type expander anchored to deciduous teeth, *SD* standard deviation

^a^Significance level *p* < 0.05 (*)

As reported in Table 5, no statistically significant differences were found by comparing groups.

**Table 5:** ANOVA and post-hoc results for inter-group comparisons

Increment T1-T0	HX-6	HX-E	HS-E
Variables	Mean ± SD	Mean ± SD	Mean ± SD
Maxillary inter-molar width (mm)	5.34 ± 2.06	3.71 ± 1.52	4.34 ± 2.33
Nasal floor width (mm)	3.05 ± 0.97	3.10 ± 2.20	2.90 ± 2.31
Nasal wall width (mm)	2.36 ± 0.99	2.45 ± 1.07	2.67 ± 2.12

*HX-6* Hyrax-type expander anchored to permanent teeth, *HX-E* Hyrax-type expander anchored to deciduous teeth, *HS-E* Haas-type expander anchored to deciduous teeth, *SD* standard deviation

The mean increase of nasal floor width was 3.05 mm for HX-6 group, 3.10 mm for HX-E group and 2.90 mm for HS-E group. In all groups these values were found to be greater than the mean increase of nasal wall width, that were respectively 2.36, 2.45 e 2.67 mm. The greatest value of expansion of maxillary inter-molar width was reported for HX-6 group (5.34 mm), followed by HS-E group (4.34 mm) and HX-E group (3.71 mm).

## Discussion

Previous studies, assessing the effects of RME on airway morphology and function, showed an increase of nasal size when maxilla was expanded [4–6, 26].

The purpose of this study was to evaluate the response of nasal cavities to three different types of expander, anchored to the permanent or deciduous teeth. No authors compared the impact of RME appliances anchored to deciduous teeth with traditional RME appliances anchored to permanent teeth in determining nasal changes. In this investigation no statistically significant differences of the variables understudy were found when RME appliance was anchored on deciduous or permanent teeth. It allows speculating that RME by using deciduous anchorage is effective, as much as traditional RME, into obtaining an increase of nasal size [18–20]. This positive impact on nasal cavity could be included in the list of the benefits previously reported by literature for this kind of anchorage, such as the lack of sequelae on permanent teeth [12, 15, 16] and the better and more stable expansion of the anterior area of maxilla with the improvement of the anterior alignment [13, 16, 17]. In the current study the differences of the nasal size increments obtained by using the modified Hyrax-type appliance (HX-E group) and the modified Haas-type appliance (HS-E group), both anchored to deciduous teeth, were no statistically significant. This outcome is consistent with the findings by Garib et al. [10] that compared tooth-borne with tooth-tissue-borne expanders anchored to permanent teeth.

Significant values of expansion were observed in all groups by studying both nasal floor width and nasal wall width. The increase of nasal floor width is comparable with that of previous studies [10]. An amount of expansion of 2.8 mm, close to the results of the current research, was recorded by Izuka et al. [37] in a CBCT study. Other authors, even finding significant enlargement of the nasal floor, did not achieve similar values of expansion [6]; the different amount of expansion applied on the patients should be taken into account to explain these discrepancies. Anyway the mentioned authors used permanent teeth as anchorage. The amount of expansion of nasal wall of the current paper is also comparable with previous studies [6, 38]. The mean increase of nasal wall width after RME was found to be lower than the mean increase of nasal floor width. This data would seem to support the reverse ‘V’ shape opening model of the cranio-facial complex on the coronal view [39].

With regards to the increase of maxillary inter-molar width achievable by using deciduous teeth as anchorage, the results of the current research revealed mean values ranged from 3.71 mm (HX-E group) to 4.34 mm (HS-E group), which are lower if compared to those reported by Cozzani et al. [14]. The authors, expanding on deciduous teeth, produced a permanent first molar expansion of 5.7 mm in cross-bite patients. Probably the different method of assessing maxillary inter-molar width changes could explain the discrepancy, since using the center of the molar fossa on dental casts could implicate a greater influence of the dental inclination on the measurement. However the maxillary molar expansion achieved in the present study could be considered clinically adequate. The difference between the two groups HX-E and HS-E appears clinically and statistically no significant.

By analysing the different kind of anchorage (i.e. permanent teeth vs deciduous teeth) with regards to the inter-molar width measurements, no significance difference was found. The lower mean values found in HX-E and HS-E group compared with HX-6 group, could be related to the lack or reduction of the buccal tipping of the permanent first molars during RME with deciduous teeth as anchorage, since no direct force was applied on them [15]. However no specific measurements about the molar crown inclination were performed in the current investigation.

CBCT and low dose CT were described in literature as accurate tools for studying cranio-facial district [21, 29] and were used both to analyse nasal cavities [18, 26, 40]. Despite this, it is relevant to underline that the use of different imaging systems in the present study might have compromised its reliability; moreover the analysis of 2D measurements by using a 3D imaging techniques could represent a limitation of the investigation.

The different clinical protocol of expansion used in the groups understudy and the lack of breathing tests to confirm anatomical and functional correlations of expansion should be considered other limits of the present study.

RME in early mixed dentition by using deciduous teeth as anchorage represents an effective treatment option for growing patients showing maxillary constriction, with potential benefits for nasal skeletal expansion. Anyway further researches would be needed to give information on the above mentioned functional consequences as well as on the long-term stability of the airway changes produced by RME on deciduous teeth. In fact significant expansion of nasal cavity shape does not assure an improvement of the breathing function which would need to be evaluated with different quantitative methods such as acoustic rhinometry.

## Conclusions

According to the results of the present investigation the following conclusions might be drawn:

- RME produces a significant skeletal transverse expansion of the nasal region in growing patients
- No significant differences in nasal effects are expected when the appliance is anchored onto deciduous teeth, with or without the palatal acrylic coverage of the expander.

## Abbreviations

ANS: Anterior nasal spine
CBCT: Cone beam computed tomography
CT: Computed tomography
CVM: Cervical vertebral maturation
HS-E: Modified Haas-type expander anchored to deciduous teeth
HX-6: Hyrax-type expander anchored to permanent teeth
HX-E: Modified Hyrax-type expander anchored to deciduous teeth
LFS: Left foramen spinosum
LMPC: Left molar pulp chamber
LNF: Left nasal floor
LNW: Left nasal wall
PNS: Posterior nasal spine
RFS: Right foramen spinosum
RME: Rapid maxillary expansion
RMF: Right molar furcation
RMPC: Right molar pulp chamber
RNF: Right nasal floor
RNW: Right nasal wall
